# Inactivation of Spores and Vegetative Forms of *Clostridioides difficile* by Chemical Biocides: Mechanisms of Biocidal Activity, Methods of Evaluation, and Environmental Aspects

**DOI:** 10.3390/ijerph19020750

**Published:** 2022-01-10

**Authors:** Weronika Augustyn, Arkadiusz Chruściel, Wiesław Hreczuch, Joanna Kalka, Patryk Tarka, Wojciech Kierat

**Affiliations:** 1 MEXEO-Wiesław Hreczuch, Energetyków 9, 47-225 Kędzierzyn-Koźle, Poland; weronika.augustyn@mexeo.pl (W.A.); mexeo@mexeo.pl (W.H.); 2 Environmental Biotechnology Department, Silesian University of Technology, Faculty of Power and Environmental Engineering, 44-100 Gliwice, Poland; Joanna.Kalka@polsl.pl; 3 Department of Social Medicine and Public Health, Medical University of Warsaw, 02-007 Warszawa, Poland; patryk.tarka@wum.edu.pl; 4 Department of Digital Systems, Silesian University of Technology, 44-100 Gliwice, Poland; wojciech.kierat@polsl.pl

**Keywords:** chlorine dioxide, disinfection, sporicidal activity, *Clostridioides*, *Clostridium difficile*

## Abstract

*Clostridioides difficile* infections (CDIs) are the most common cause of acquired diseases in hospitalized patients. Effective surface disinfection, focused on the inactivation of the spores of this pathogen, is a decisive factor in reducing the number of nosocomial cases of CDI infections. An efficient disinfection procedure is the result of both the properties of the biocidal agent used and the technology of its implementation as well as a reliable, experimental methodology for assessing the activity of the biocidal active substance based on laboratory models that adequately represent real clinical conditions. This study reviews the state of knowledge regarding the properties and biochemical basis of the action mechanisms of sporicidal substances, with emphasis on chlorine dioxide (ClO_2_). Among the analyzed biocides, in addition to ClO_2_, active chlorine, hydrogen peroxide, peracetic acid, and glutaraldehyde were characterized. Due to the relatively high sporicidal effectiveness and effective control of bacterial biofilm, as well as safety in a health and environmental context, the use of ClO_2_ is an attractive alternative in the control of nosocomial infections of CD etiology. In terms of the methods of assessing the biocidal effectiveness, suspension and carrier standards are discussed.

## 1. Introduction

The One Health approach recognizes that human health is closely connected to the health of animals and the natural environment. The paradigm of One Health is based on the observation that health is a continuous, combined, global, and interdependent series of causes and effects occurring within ecosystems as well as in human and animal populations [1,2,3]. Therefore, the One Health approach refers to the protection of human health in line with environmental protection. This approach has become increasingly important against the background of the ongoing global pandemic. It is worth noting that in the last three decades, more than 70% of infectious human diseases were zoonoses [3]. These are diseases whose etiological factors originate from farm and companion animals, as well as wild animals. New zoonoses have recently appeared more frequently, especially in the reservoirs of microorganisms occurring in nondomestic animals [4,5]. The factors favoring the appearance of unknown zoonoses include constant changes in ecosystems and local environments. An important topic in the context of the One Health approach is antibiotic resistance, as several habitats may be sites for the emergence and maintenance of resistant microorganisms, including hospital effluents, wastewater treatment plants, farms, or aquaculture ponds [6]. In such habitats, genetic material that determines antibiotic resistance is transferred to the genomes of antibiotic-sensitive bacteria.

To face the challenges of a changing world, intensive cooperation among veterinarians, medical doctors, environmentalists, chemists, and biologists is crucial. Emerging new infections require action to develop effective drugs, but also special precautions to prevent the spread of infections. It should also be noted that an equally important issue in contemporary infectiology is the existence of species that have managed to develop over the course of their evolution effective mechanisms of high resistance to biocides. These include enteropathogenic bacteria of the *genera Salmonella*, *Campylobacter*, and *Clostridium*, as well as *Escherichia coli*, which are the cause of food toxico-infections [7]. The development of new, effective disinfection methods is important not only in medical areas, but also in processes related to environmental engineering and securing access to drinking water.

In North America and Europe, infections with *Clostridioides difficile* (CDI) are the most common causes of intestinal diseases acquired during hospitalization. This phenomenon is associated with the use of antibiotics and chemotherapy, as well as with ineffective decontamination of the hospital environment [8]. On average, CDI occurs in 4–8 of 1000 hospital patients [9]. In this regard, over 1 million infections are recorded in the USA alone [10], with about 29,000 deaths per year, of which more than 80% occur in immunocompromised patients over 65 years of age [11,12]. The cost of US health care related to the fight against CDI is about $ 6 billion per year [13]. In Germany, in 2019, over 2200 cases of CDI with serious clinical condition were reported, with 20% having a fatal outcome [14]. It was calculated that the average German hospital will treat approximately 96 cases of CDI each year; 70% of them will be acquired during hospitalization [15]. According to the 2016 Annual Epidemiological Report of the US Department of Health and Human Services (CDC 2018), the highest hospitalization-related morbidity rates, with an average rate of 2.38 per 10,000 hospitalized person-days, have been reported for Estonia (12.93), Lithuania (7.88), and Poland (6.18). Germany did not participate in these studies.

The bacterium *Clostridioides difficile* (CD) was first isolated in 1935 from the stool of a healthy infant [16]. In the 1970s, it was shown to be associated with post-antibiotic diarrhea, pseudomembranous colitis, and intestinal perforation [17]. It belongs to the group of gram-positive anaerobic rods, forming spores that enable long-term survival in the external environment [18]. This pathogen produces two toxins, namely *Clostrioides difficile A* (TcdA) toxin and *Clostrioides difficile B* (TcdB) toxin, which are large multidomain proteins with a molecular weight of 308 and 270 kDa, respectively [19]. The CD strain of the R027 ribotype is particularly difficult to combat and, unlike others, produces several times more TcdA and TcdB, as well as a binary toxin with ADP-ribosyltransferase activity, involved in cell apoptosis and contributing to significant tissue damage [20,21].

According to the latest estimates, more than half of the CDI cases are associated with healthcare facilities [22]. Since CD is transmitted by the fecal-oral route through contaminated hands, the proper strategy to fight this microorganism is the cleaning and disinfection of sanitary surfaces and frequently touched surfaces [23]. Because of the high resistance of spores to chemical disinfection, many washing and disinfecting agents commonly used in hospitals, including alcohol-based agents, are not effective against CD [24], making the use of specialized preparations mandatory. For manual surface cleaning, products based on active chlorine, glutaric aldehyde, ortho-phthalic aldehyde, and peracetic acid are often used [25]. Among the non-contact disinfection methods, gaseous hydrogen peroxide is used in automatic processes [22]. To increase the efficiency of disinfection, mixed techniques are also used, such as the application of an oxidant and UV light [26,27]. Later in the article, we discuss the mechanism of the biocidal action of the chemical compounds most commonly used for disinfection in healthcare facilities.

## 2. Characterization of Biocides Against *Clostridioides difficile* Used in Healthcare Settings

### 2.1. Active Chlorine

The commonly used term “active chlorine”, adopted from the terminological area of the analytical chemistry of sodium chlorate (I) solutions, is used to refer to preparations containing chlorine with oxidation states 0 and +1, in contrast to chloride ions Cl^−^ without biocidal properties. Application forms of preparations based on active chlorine are usually aqueous solutions containing a complex, equilibrium system of several forms of chlorine, with concentrations resulting from the pH of the solution, i.e., elemental chlorine (Cl_2_), chloric acid (HClO), chlorate ions (I) (ClO^−^), as well as small amounts of complex trichloride (Cl^3−^) ions [28,29]. In view of the complexity of the issue, the chemistry of the above systems requires a wider discussion.

Active chlorine solutions used in disinfection are generally obtained from preparations containing active chlorine-releasing substances (CRAs, chlorine-releasing agents), including sodium hypochlorite (formerly “sodium hypochlorite”) and selected *N*-chlorine derivatives of amines, such as sodium dichloroisocyanurate (NaDCC) or chloramines [30,31].

Commercially available sodium hypochlorite solutions are produced by absorption of elemental chlorine in sodium hydroxide solution (1):

(1) $$Cl_{2}+2\;NaOH⇋NaClO+NaCl+H_{2}O\;$$

A by-product of Reaction (1) is sodium chloride, some amounts of which are present in sodium hypochlorite solutions, also due to the partial decomposition of sodium hypochlorite (2):

(2) NaClO⇋NaCl+12H2O

Under the conditions of an excess of hydroxide ions (pH > 7) in an aqueous solution, sodium chlorate (I) undergoes electrolytic dissociation according to Equation (3):

(3) $$NaClO⇋Na^{+}+ClO^{-}\;$$

Lowering the content of hydroxyl ions leads to the production of poorly dissociated chloric acid (I) (4), considered as a form of active chlorine, which mainly determines the biocidal properties [30] (4):

(4) $${\mathrm{ClO}}^{-}+H_{2}O⇋\mathrm{HClO}+{\mathrm{OH}}^{-}$$

Further reduction of the pH of the solution leads to the opposite reaction (1) and the production of molecular chlorine.

**Figure 1:**
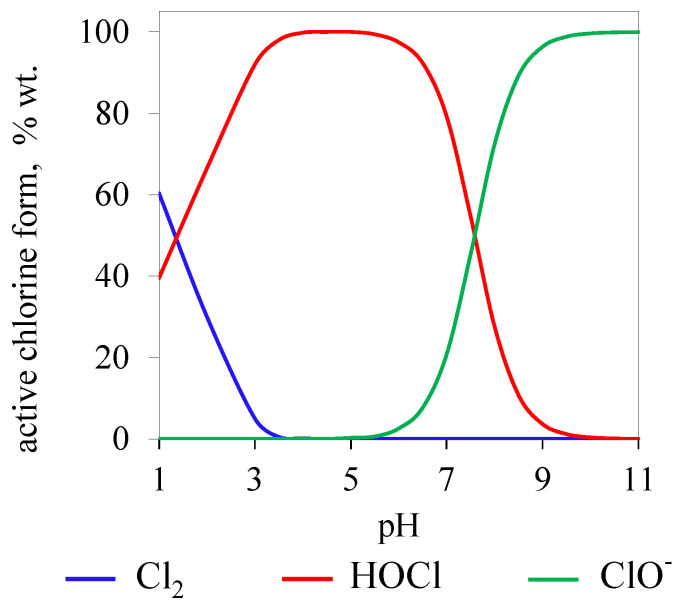
Curves of equilibrium concentrations of elemental chlorine (Cl_2_), chloric acid (HOCl), and chlorate (I) ions (ClO^−^) in aqueous solutions depending on the pH.

Figure 1 shows the equilibrium forms of active chlorine in a solution of sodium hypochlorite, depending on the pH of the solution, based on the data presented by the Black & Veatch Corporation [32].

The course of the equilibrium curves presented in Figure 1. shows that the maximum concentration of biocidal chloric acid (I) occurs between pH 3 and 6. Below this range, the HOCl concentration drops at the expense of the production of molecular chlorine. In solutions with pH > 7, the share of ClO^-^ ions with lower biocidal effectiveness increases.

In the case of using *N*-chlorinated derivatives, the source of chloric acid (I) is the hydrolysis reaction of sodium diisocyanurate or chloramine, according to Equations (5) and (6). As mentioned earlier, HOCl mainly determines the biocidal properties.




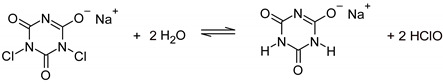

(5)






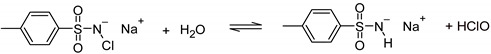

(6)



The biocidal mechanism of chloric acid (I) action on microbial cells consists of the disruption of metabolic pathways essential for the functioning of the cell and the degradation of genetic material. The influence of HOCl from chloramine (NH_2_Cl) has been investigated as early as in the 1970s. Authors Shih and Lederberg [33] showed the effect of a significant decrease in the transformational capacity of *B. subtilis* DNA under the influence of several dozen µmol/L HOCl solutions. Studies conducted with the use of isolated *E. coli* RNA nucleotides (CMP, AMP, GMP) showed significant changes in their molecular structure, confirmed by UV spectrophotometry. Other authors [34] showed that HOCl can undergo a Fenton-type reaction, leading, similar to H_2_O_2_, to the production of HO· radicals, the interaction of which with DNA leads to degradation.

The strong influence of chloric acid (I) on oxidative phosphorylation pathway disruption as well as on other processes involving the cell membrane has been demonstrated [35,36]. The effect of 96% inhibition of DNA synthesis and complete inhibition of E. coli growth was found at a concentration of 2.6 mg/L HOCl [37].

The most frequently used preparations containing active chlorine with sporicidal properties are sodium hypochlorite [38] and sodium dichloroisocyanurate (NaDCC) [39]. However, sporicidal activity requires higher concentrations (for NaClO above 200 mg/L, *N*-chloro derivatives > 1000 mg/L) [40,41,42].

The decisive stage in the mechanism of the sporicidal action of active chlorine seems to be the increase in the permeability of the spore coating (cortex), manifested by the release of dipicolinic acid (DPA) [43]. The mechanism of the sporicidal activity of HClO is also based on the degradation of the structures of peptides and amino acids, lipids, and nucleic acids in pathogen cells [44]. In studies of 21 CD isolates, 1000 mg/L of active chlorine reduced spores by L = 4-6-LOG during a contact period of 10 min, depending on the clinical strain. The equation is as follows:

(7) $$L=\log_{10}\frac{A}{B}$$

where A is the microbial count before decontamination and B is the microbial count after decontamination.

Lowering the concentration to 500 mg/L resulted in the reduction of the most infectious CD isolate ribotype R027 to L = 1.2–2.4-LOG. In addition, the use of active chlorine concentrations below the recommended levels may affect the hydrophobicity of spores and, thus, their ability to adhere to the surface, which in turn may favor the spread of the pathogen [45]. However, preparations containing active chlorine show a number of disadvantages: a destructive effect on rubber and plastics, as well as metal corrosion. They are also unstable, and their decomposition can be accelerated by solar radiation and elevated temperatures.

A significant environmental issue related to the use of preparations based on active chlorine is their ability to chlorinate organic matter and produce disinfection by-products (DBPs), which potentially harm the environment and human health [46,47]. Previous results [48,49,50,51] show that chlorination or chloramination in water environments containing amino acids or humic substances derived from soil can result in the production of the toxic substances cyanogen chloride, dichloracetonitrile, and organochloramines.

### 2.2. Glutaraldehyde

The critical stage in the mechanism of the biocidal action of aldehydes is the denaturation of proteins and the degradation of RNA and DNA [52]. This is the effect of the high reactivity of the aldehyde group, in particular with respect to amino groups, leading to irreversible condensation with the formation of a stable imide linkage (8). The result is an irreversible loss of functionality of the biochemical particles with amine groups [53].




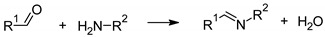

(8)



The most popular aldehyde to combat CD is glutaraldehyde (GA). The mechanism of action of bifunctional glutaraldehyde is the formation of imide bonds with lipopeptides and peptidoglycan of bacterial cell walls, causing their deformation. Glutaral also reduces dipicolinic acid formation, which is characteristic of spores and determines their external factor resistance [54]. The advantage of products based on glutaraldehyde is the low price, but they can denature proteins, which may be dangerous in the case of disinfection of surfaces susceptible to biofilm formation. Moreover, GA is one of the substances that are an odor nuisance, toxic, and dangerous for the natural environment, further reducing its value [55].

Glutaraldehyde has a high sporicidal activity. A 1% GA solution, with a contact period of 15 min, can reduce CD spores at the L = 1.03-LOG level, and a 6% solution can reduce such spores at the L = 2.05-LOG level. The discussed results were obtained using the “four-field” method according to the EN 16615 standard [56]. However, the spore reduction of L ≥ 4.0-LOG, required in the medical field, was not achieved [15]. Products based on GA are most frequently used in the disinfection of medical instruments and equipment, especially in endoscope disinfection. However, compared to peracetic acid or *o*-phthalaldehyde, the disinfection results were poorer [25]. In this sense, the use of GA-based formulations for CD appears controversial.

### 2.3. Hydrogen Peroxide

Hydrogen peroxide (H_2_O_2_) is a strongly oxidizing, odorless substance with strong biocidal properties in liquid and gaseous form. Low concentrations of gaseous H_2_O_2_ are more effective than liquid forms [57]. The mechanism of action is based on the production of highly reactive hydroxyl radicals (9), oxidizing thiol groups in proteins, lipids, and nucleic acids [58]. Its rapid decomposition into oxygen and water reduces the toxicological hazards associated with its use.

(9) $$H_{2}O_{2}+e^{-}+H^{+}\to H_{2}O+{\mathrm{OH}}^{\cdot }$$

The biocidal activity of preparations with H_2_O_2_ increases with increasing temperature [59]. The L = 4-LOG reduction level for *B. subtilis* spores can be achieved within 11 min for a 26% solution. Increasing the temperature to 76ºC allows achieving the same reduction within 30 s [60]. Treatment of CD spores with 0.5% H_2_O_2_ wipes for 30 s and 3 min reduced the pathogen by L = 2.8 [61].

Techniques of surface disinfection with gaseous H_2_O_2_ in automatic disinfection processes have been developed and commercialized in the 1980s and 1990s [62]. There are three basic disinfection systems using H_2_O_2_: HPV (hydrogen peroxide vapor) by Bioquell, VHP (vapor hydrogen peroxide) by Steris, and aHP (aerosolized hydrogen peroxide) by Sterinis [58,63].

The HPV and aHP systems have been tested in the decontamination of hospital wards affected by CDI. The HPV system introduced to hospitals for two periods of 10 months reduced the amount of CDI by 53% compared to the control [64]. In the case of the aHP fogging system, its disinfection efficiency was compared to that of sodium hypochlorite. Efficiency was obtained for 91% of hospital rooms where people infected with CD were present. Fogging was carried out for 1 h using a 5% solution of H_2_O_2_ with the addition of a silver cation. In the case of 0.5% sodium hypochlorite solution (5 mg/L active chlorine), the effectiveness of the disinfection process was noticed in 50% of the disinfected rooms. By testing the disinfection effectiveness of the same products in vitro on polyvinyl chloride or laminate carriers, imitating floors and furniture in hospital rooms, spore reductions of L = 4.18 ± 0.8-LOG for H_2_O_2_ solution and L = 4.32 ± 0.35-LOG for sodium hypochlorite were obtained [65].

Decontamination with H_2_O_2_ largely depends on the type of the disinfected surface. Significant differences were found in the effectiveness of decontamination of *B. subtilis* spores, depending on surface porosity. Within 20 min and at a concentration >1000 mg/L H_2_O_2_ on porous surfaces of industrial flooring and pine wood, the level of pathogen reduction, respectively, L = 1.6-LOG and L = 2.2-LOG. On non-porous glass or cardboard surfaces, spore reduction levels were L = 7.5-LOG and L = 7.6-LOG [66].

### 2.4. Peracetic Acid

Peracetic acid (PAA) is a colorless, clear liquid with an acetic odor. It is a strong oxidant, with an oxidation potential comparable to that of ozone [67].

The essence of PAA’s biocidal activity lies in its ability to homolyze, with the formation of highly reactive hydroxyl radicals (10) [68]:




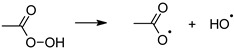

(10)



These, in turn, interact with organic matter, analogously to hydrogen peroxide. The advantage of peracetic acid over H_2_O_2_ is its resistance to decomposition by peroxidases, which allows it to remain active [52]. As a result of the reaction of free radicals with amino acids, biological proteins are inactivated, leading to inhibition of the vital functions of pathogens. In this sense, PAA is an alternative to GA products. However, the stability of its working solutions is low, it has a strong odor, and can corrode surfaces [60].

In the EN 16615 carrier method against CD, the expected reduction of L ≥ 4-LOG spores was obtained during a contact period of 15 min for a solution with a PAA concentration of 4% [15]. In endoscope disinfection, the use of PAA resulted in shorter exposure times than required for GA or ortho-phthalaldehyde. The reduction of CD in the endoscope after a contact period of 5 min at 30 °C was L ≥ 5-LOG [25].

### 2.5. Chlorine Dioxide

**Figure 2:**
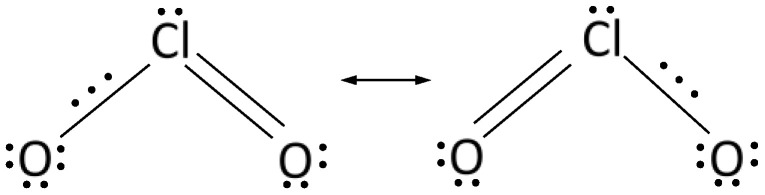
Electronic structure of the chlorine dioxide molecule.

Due to the lack of the ability to chlorinate organic matter and, thus, to produce ecotoxic chlorine-based disinfection byproducts, ClO_2_ is an alternative to commonly used preparations based on active chlorine and other biocides. The strong oxidizing properties of ClO_2_, combined with the selectivity of the biocidal action, and the inability to form harmful by-products of disinfection justify the increased interest in this chemical compound as an active ingredient in disinfectants [32,69]. The above-mentioned specific properties of ClO_2_ directly result from its unusual molecular structure, presented in the form of resonance structures in Figure 2, proposed by Pauling [70].

The ClO_2_ molecule is a free radical where the unpaired electron is not located at a specific atom but is part of a covalent three-electron bond. This explains the stability of the ClO_2_ molecule and the lack of a tendency to dimerize to form Cl_2_O_4_, unlike other simple inorganic radicals with similar symmetry. The ClO_2_ is a yellow-brown gas with a characteristic acrid odor. At 1 atm pressure, ClO_2_ boils at 11 °C and melts at −59 °C. At higher concentrations, both gaseous and liquid ClO_2_ have strong explosive properties. Hence, in practical use, ClO_2_ is not compressed but generated in situ in aqueous solutions at concentrations not exceeding 3 g/L. It can also be released as a gas at concentrations below the explosive limit [71].

Studies on the chemical activity of ClO_2_ in aqueous solutions have shown the possibility of effective and quick reactions with various functional groups by free radical oxidation [72,73,74,75,76].

The non-specific mechanism of the bactericidal and virucidal action of ClO_2_ can therefore be explained by the reactions with numerous molecules of key importance for the organism under attack, at different levels of the cell structure (cell membrane, organelles, intracellular processes) and virus (protein envelope, genetic material). Because of its strong oxidizing properties, ClO_2_, especially in aqueous solutions, exhibits biocidal activity by influencing the cellular equilibrium of electron transfer processes.

The mechanism of protein denaturation via ClO_2_ is based on a reaction with six amino acids: cysteine, tryptophan, tyrosine, proline, hydroxyproline, and histidine; reactivity with the first three is extremely high [77].

In the case of cysteine, degradation and loss of biochemical functions is the result of dimerization of this amino acid, with the formation of a disulfide bond [78]. The precursor of the reaction is a thiyl radical formed as a result of the high susceptibility of the thiol group of cysteine to radical oxidation [79]. Recombination of the thiyl radical with another ClO_2_ molecule gives a transient Cys-ClO_2_ adduct. At high pH values, reaction with another cysteine thiol radical leads to the formation of the aforementioned dimer (cystine); at a low pH, cysteic sulfonic acid is generated (11) and (12) [80].




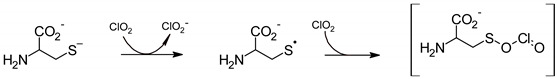

(11)






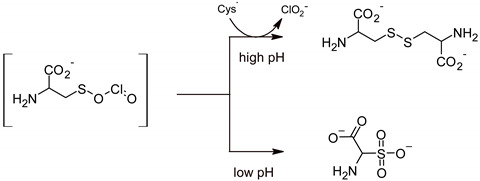

(12)



An example of the degradation of an amino acid under the influence of ClO_2_ with the participation of amine nitrogen atoms is the production of a radical precursor of tryptophan oxidation. The diagram shows one of the two alternative pathways of the radical attack of the ClO_2_ molecule directed at the indole nitrogen atom, leading to the formation of a radical cation and, after proton cleavage, a resonance-stabilized radical, the extreme mesomeric forms of which are shown in Equation (13) [81]:




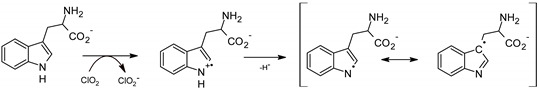

(13)



The radical presented in the above scheme is a precursor in the reaction of formation of among others *N*-formylokinurenine, formed as a result of the attack of the ClO_2_ radical on the 3-yl carbon atom of the indole moiety [82]. Under the influence of ClO_2_, tyrosine is oxidized to 3,4-dihydroxyphenylalanine (DOPA) or 2,4,5-trihydroxyphenylalanine [83].

One interesting finding, shedding light on the possible mechanism of the influence of ClO_2_ on living organisms, is the strong ClO_2_ interference in redox processes, determining electrochemical equilibria in mitochondria and cell membranes, in particular with regard to the NADH/NAD + redox system responsible for cellular respiration and mediation in ATP synthesis [84,85]).

**Figure 3:**
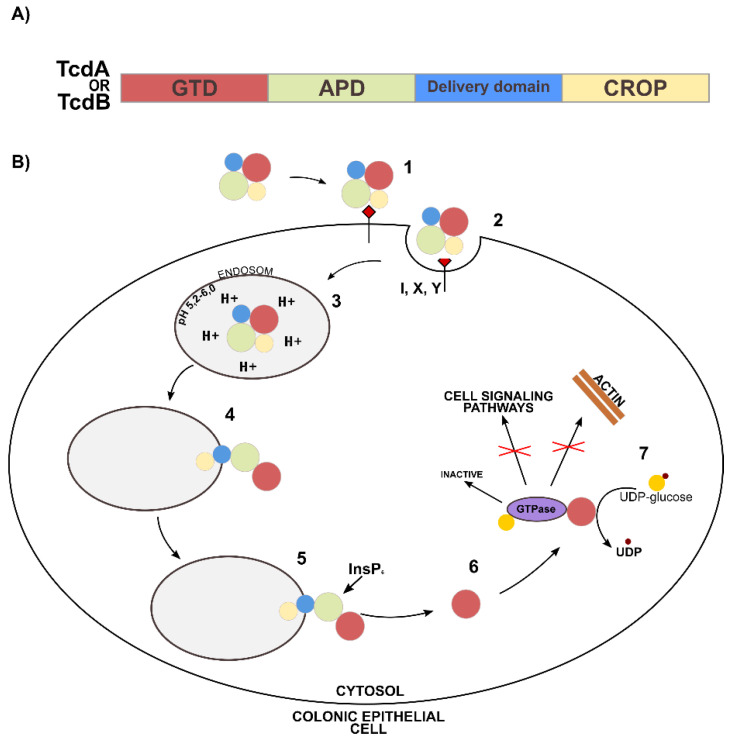
Mechanism of action of TcdA and TcdB. (**A**) Four functional domains of TcdA and TcdB: glycosyltransferase domain (GTD), autoprocessing domain (APD), translocation domain, repeat oligopeptide domain (CROP,) (**B**) Multistage mechanism of cell intoxication by TcdA and TcdB [87].

In the context of amino acid composition, it is worth analyzing the potential mechanism of CD toxin inactivation against ClO_2_. At the amino acid level, TcdA and TcdB are approximately 45% identical [86]. They have a similar multi-domain structure, consisting of a glucosyltransferase (GTD) domain, a processing domain (APD), a translocation domain, and a C-terminal domain (CROP) [87]. Both TcdA and TcdB, due to repeating oligopeptides of the CROP domain, bind to receptors on the cell surface. The TcdA receptors can be carbohydrate antigens designated as I, X, Y, occurring in the human intestinal epithelium (Figure 3B—step 1) [88]. After endocytosis (Figure 3B—step 2), toxins penetrate the endosomes (Figure 3B—step 3), where low pH values facilitate conformational changes in the translocation domain, exposing hydrophobic residues and creating a channel through which GTD domains and, most likely, APD (Figure 3B). Figure 3B—step 4 enter the cytosol. The process of GTD cleavage and release is induced by the APD domain, to which inositol hexakisphosphate (InsP6) binds (Figure 3B—step 5) [87,89,90]. The released GTD domain inactivates intracellular GTPases from the Rho and Ras families (Figure 3B—step 6). Glycosylation, i.e., the transfer of glucose from UDP-glucose to threonine-37, occurs in the Rho protein and threonine-35 (in the Rac and Cdc42 proteins) [91,92]. This blocks the biological functions involved in regulating the actin cytoskeleton and disrupting cell signaling (Figure 3B—step 7) [86], resulting in a cytopathic effect, rounding, and necrosis of the host cells.

According to the available literature, the interaction of ClO_2_ with CD can therefore take place on several levels. The APD auto-processing domain is a protease containing cysteine and histidine residues in the active center, most likely responsible for the binding of zinc ions, facilitating the proper release of the GTD domain into the cytosol [93]. At the catalytic center of GTD, there is a tryptophan residue which binds UDP-glucose. Tryptophan is a key amino acid determining enzymatic activity and, thus, the cytotoxicity of large CD toxins [94]. Oxidation of the aforementioned amino acids with ClO_2_ may influence the key stages of the TcdA and TcdB mechanisms in the cell and, thus, inhibit the cytotoxic effect of host proteins.

The possible paths of ClO_2_ reactions with elements of biologically active molecule structures presented above are mainly the result of studies using isolated, pure forms of biologically active target compounds. Unfortunately, reports describing the results of studies on the mechanisms of biocidal activity in vivo, based on observations of changes in living organisms, are few, and the obtained conclusions are not consistent. For example, the results of Zhu et al. indicate that ClO_2_ in concentrations higher than 100 mg/L damages DNA in *Saccharomyces cerevisiae* [95]. This does not confirm DNA damage as the primary cause in the biocidal action mechanism as reported in a previous study [96]. Similarly, ambiguities are observed in the investigations of the mechanism of sporicidal action of ClO_2_. Young and Setlow reported that *B. subtilis* spores treated with ClO_2_ showed no tendency to accumulate DNA damage [97]. The occurrence of the above effect was found both in spores in which low-molecular-weight DNA protects proteins against genotoxins (SASP) and strains lacking the sspA genes and sspB coding for SASP proteins were produced; there were no symptoms of mutagenesis under the influence of ClO_2_.

Increased sensitivity to ClO_2_ was observed for spores lacking the coat protein, examined with chemical methods or by mutation of genes encoding respective proteins. Although no release of dipicolinic acid (DPA) was found in the ClO_2_-treated spores; the spores released DPA much more easily after sub-lethal heat treatment than untreated ones. The above observations lead us to the conclusion that the critical process of the mechanism of action of the sporicidal ClO_2_ solution against *B. subtilis* spores is not DNA damage, but the damage to the cortex layer composed of, among others, DPA.

An attempt to create a molecular basis for explaining the mechanism of the sporicidal action of ClO_2_ has been described elsewhere [98]. The study focused on the observation of the expected changes in the chemical structure of DPA and DNA as well as selected amino acids of *Bacillus thuringiensis* spores exposed to the ClO_2_ solution with a concentration of 750 mg/L. The study of molecular structure changes was carried out using Raman spectroscopy and the optical laser tweezer (LTRS) technique. Changes in the structure of spores were determined via scanning electron microscopy (SEM) and transmission electron microscopy (TEM). Although a high degree of spore reduction was observed under the influence of the ClO_2_ solution (5-6-LOG was obtained), no visible changes were found in the Raman spectra, both for the bands characteristic for DPA and DNA as well as for amide bands related to possible changes in the structure of amino acids. There were also no significant changes in the SEM and TEM images of the spores under the influence of ClO_2_.

Although studies on the effectiveness of ClO_2_-based preparations are scarce, the estimated sporicidal properties of ClO_2_ should be considered attractive, in particular in relation to the low risks to human health and the environment. Foegeding et al. [99] studied the effects of ClO_2_ on the survival of *Bacillus cereus* T, *B. cereus* F4810/72, Bacillus stearothermophilus ATCC 1518, and *Clostridium perfringens* NCTC 8798 spores in the environment of a relatively low concentration of ClO_2_ (20–80 ppm) at pH 4.5 and 8.5, using various sporulation methods.

Comparison of sporicidal properties showed the effect of *B. cereus* T sporulation method on survival. In the case of *C. perfringens* spores, a decrease in survival was observed at higher pH. At a concentration of 50 mg/L, the level of 4-LOG reduction at pH 4.5 was achieved for 15–30 min for *B. cereus* spores (depending on the sporulation method), 20 min for *B. subtilis*, and 80 min for *C. perfringens*. The same level of reduction at pH 8.5 was achieved after 45 min for *C. perfringens*. The influence of the pH on the survival of the remaining spores was insignificant. In another study [100], spores of *B. pumilus* SAFR-032 and *B. subtilis* ATCC 6051 model strains for *C. difficile*, resistant to disinfection with hydrogen peroxide, under unloaded conditions and in ClO_2_ aqueous solutions were investigated. For the concentration of 47 mg/L and period of 10, 60, and 24 min, reduction levels of 1-LOG and 2-LOG were obtained, respectively. Complete reduction was only achieved after 24 h. However, increasing the ClO_2_ concentration to 187 mg/L achieved complete spore reduction within 10 min. In 2005, Perez et al. demonstrated that a solution with a concentration of 600 mg/L ClO_2_ reduced CD spores to the level of L = 6 LOG within 15–30 min, depending on the strain and the condition of the microorganisms. The tests were carried out in unloaded (clean) conditions with the use of steel disc tests [101]. In another study, reduction of CD at the level of 2.48-LOG and 2.65-LOG was achieved for the concentration of 1,600 mg/L ClO_2_ during 10 min without organic load on sterile glass carriers [102].

The suspension tests of the authors of this publication, carried out in accordance with the EN 17126 standard, showed the effectiveness in controlling CD at the level of 4.49-LOG for a solution with a concentration of 100 mg/L ClO_2_ under clean conditions and a contact period of 5 min.

## 3. Standardized Methods for Assessing the Sporicidal Activity of Disinfectants

As already mentioned, an important element in the control of CDI is surface disinfection, especially the so-called “tactile”. Producers of disinfectants demonstrate their sporicidal activity through suspension and/or carrier tests.

### 3.1. Suspension Methods

Until 2018, the standards EN 14347:2005 and EN 13704:2018-09 [103,104] were available. The first one covers Phase 1, Step 1 studies, in which, according to the provisions of the European Committee for Standardization (CEN), it is determined whether the active substance exhibits a sporicidal effect at the stage of selecting the product’s formula ingredients. The standard applies to products used in agriculture, home and service hygiene, food, as well as in the industry, public utilities, and medicine and veterinary medicine. The tests are carried out without any organic burden on *Bacillus subtilis* and *Bacillus cereus* spores. However, the responsible implementation of biocides requires further Phase 2 research.

The suspension standard EN 13704:2018-09 describes Phase 2, Step 1 tests, which consist of determining the effectiveness parameters, i.e., concentration and contact period under the conditions of use of the preparation. Mandatory tests are performed in clean and unclean conditions for *Bacillus subtilis* spores and for *Bacillus cereus* spores and *Clostridium sporogenes*. Products placed on the market and used in medical areas were mostly tested according to the described standard but under unclean conditions, with an increased organic load (3.0 g/L of bovine albumin and 3 mL/L of defibrinated sheep blood).

Since 2018, the suspension standard EN 17126:2018-01 [105] has been available for testing the sporicidal effectiveness of disinfectants in the medical field. It covers the use of preparations for disinfecting surfaces, tools, and textiles in two areas, sporicidal and sporicidal against CD R027 NCTC 13366. It is currently the only standard approved by the CEN that describes the methodology for determining the effectiveness of the preparation against CD. However, due to the direct contact of disinfectants with the pathogen suspension, this method still does not reflect the actual conditions of the disinfection process. Therefore, Phase 2, Step 2 studies (the so-called “carrier studies”) were carried out, in which the effectiveness was determined in conditions simulating practical application [106]. The essence of the problem is that the spore-active formulations in the suspension tests do not achieve the required reduction in the carrier tests. As a result, the recommended concentrations of the disinfectant are not effective in practice.

### 3.2. Carrier Methods

**Figure 4:**
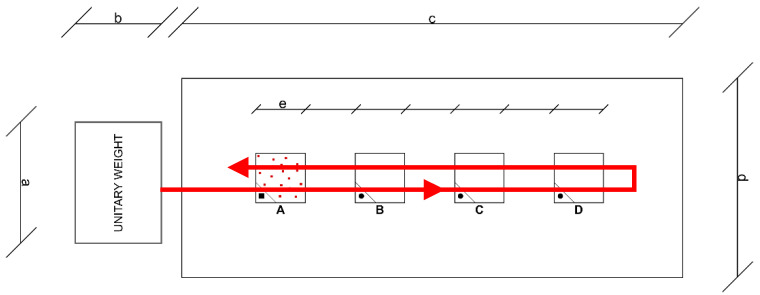
Operation diagram in the four-pole method. Test surface with four test fields (**A**–**D**) and designated wiping direction and unitary weight. a = 8.6 cm; b = 12.1 cm; c = 50 cm; d = 20 cm; e = 5 cm. **A**—contaminated test field.

Currently, there are no approved carrier standards for liquid disinfectants for the control of spores in medical areas. Some manufacturers of sporicidal disinfectants specify the effectiveness of their preparations against CD or *Bacillus subtilis* spores in accordance with EN 13697:2015 [107]. Gemein et al. [15] proposed a carrier method using the mechanical factor of sporicidal activity against CD, based on the standard EN 16615:2015, intended for the medical field. This is the so-called “four-field method”, thanks to which it is possible to test the effectiveness of microbial reduction on contaminated test surfaces and also the extent of the spread of spores [108]. The method uses a wiping mechanism, with a tissue soaked in a disinfectant, and a test surface with four designated areas, in which only the first is contaminated with spores. As shown in the Figure 4, the method takes into account the effect of secondary spread of the pathogen during rubbing.

The red polygon arrow line shows the trajectory of the disinfectant tissue, moved along the four fields, starting from infected field A through uninfected fields B, C, D, and back. After rubbing the remaining fields, the degree of pathogen spread is determined.

### 3.3. Carrier Methods for Assessing the Effectiveness of Automatic Air Disinfection

One interesting aspect in the context of the assessment of sporicidal activity is the use of the carrier standard EN 17272:2020, approved and published in 2020 [109]. The standard describes methods of disinfecting surfaces by air with the use of automated processes involving the emission of an aerosol or gas of active substances into the air through stationary (stationary) devices. Determining the effectiveness of preparations is used in the fields of medicine, veterinary, industry, and public utility. The mentioned standard applies to the processes controlling vegetative forms of bacteria, mycobacteria, fungi, viruses, and bacteriophages, as well as their spores. The obligatory strain in the assessment of sporicidal activity according to the standard is *Bacillus subtilis* ATCC 6633. In addition, it facilitates testing the effectiveness against any pathogen, including CD.

## 4. Conclusions

Biocides used in clinical disinfection for the control of bacteria resistant to disinfection treatments, in particular spore-forming bacteria, are usually simple, low-molecular-weight chemical compounds with strong oxidants (active chlorine, peroxygen compounds) or electrophiles with high affinity for atoms with non-binding electron pairs (e.g., amino nitrogen atoms).

The mechanism of the biocidal action of the above compounds mainly consists of the denaturation of proteins by generating irreversible structural changes at the level of functional groups of individual amino acids, such as:

- oxidation of thiol groups with the formation of disulfide bridges or derivatization of amine compounds by radical oxidation (oxidants);
- formation of imine bonds (Schiff compounds) by condensation of amino groups of amino acids with carbonyl groups (aldehydes).

The disruption of intracellular redox processes, which constitute steps in metabolic pathways (oxidants, in particular ClO_2_), may be an important step in the mechanism of the killing action. Despite the natural ability of spores to produce proteins that protect DNA against genotoxic effects (UV radiation, chemical genotoxins), the factor determining the resistance of spores to the action of the discussed substances, including ClO_2_ or HClO, is their protein shell (coat).

Among the discussed biocidal compounds effective in controlling vegetative forms and CD spores, one attractive alternative is chlorine dioxide, because of its high biocidal effectiveness and low risks for human health and the environment. Although the underlying mechanism has not been fully elucidated, this compound shows a relatively high sporicidal activity. The way in which it is carried out experimentally plays a key role in assessing the effectiveness of the sporicidal disinfectant.

The key condition for the reliability of the assessment of effectiveness, affecting the final test result, is the adequacy of the mapping of the real object, which is a fragment of a diverse, usually non-standard clinical environment, by artificially created model conditions of a laboratory method. The approximation of laboratory conditions to real conditions is achieved through the implementation of subsequent phases and stages of biocidal effectiveness assessment.

The suspension standard EN 17126:2018-01 is an example of the procedure in Phase 2 of Step 1 of the assessment of sporicidal activity of chemical disinfectants. The next stage of approaching the real clinical environment is the designed carrier standards in Phase 2 of Step 2. The carrier standards simulate the conditions occurring during surface disinfection; they can be divided into those with and without the use of a mechanical factor. The four-field carrier test, included in the EN 16615 standard and intended for the evaluation of sporicidal activity, allows the evaluation of the degree of spore reduction on the surface with the use of a chemical and mechanical agent, to determine whether the preparation has a prolonged effect and thus can be used for periods of up to 1 h, and estimate the effect of transferring spores to other surfaces during e disinfection. The use of a vehicle test, applying a mechanical factor will be the largest step in assessing the sporicidal activity of chemical disinfectants.

## Data Availability

Not applicable.
